# The Circular Decision-Making Tree: an Operational Framework

**DOI:** 10.1007/s43615-022-00194-6

**Published:** 2022-09-21

**Authors:** Rachel Greer, Timo von Wirth, Derk Loorbach

**Affiliations:** Dutch Research Institute for Transitions (DRIFT), Erasmus University Rotterdam, Rotterdam, Netherlands

**Keywords:** Circular economy, Decision-making, Eco-efficiency, Sustainable innovation, Diffusion potential, Sustainability transitions

## Abstract

Because of the need to limit extraction of raw materials and reduce amounts and impacts of waste, countries and businesses are challenged to transition to a circular economy: an economic system in which the materials are reduced, reused, or recycled, but not wasted. Yet, transitioning from a linear to a circular economy implies societal-level, structural changes that have deep implications for existing business models and practices–and the current economic system is still largely organized around virgin material extraction and linear modes of production and consumption. Despite stated ambitions at various geographical scales to become more or fully circular, the outcomes still fall short of such visions. One important reason why the transition towards a circular economy is not proceeding as quickly as hoped can be found in the decision processes used by companies, investors, and policy makers. Suitable frameworks that support decision-making could thus be a key enabler of this transition, if based upon a circular and transformative, rather than a linear optimization logic. In this paper, we therefore explore a different decision-making logic that is developed based on circularity. This provides the basis for an operational framework designed to help decision-makers such as policymakers, investors, and entrepreneurs navigate tradeoffs and take decisions considering the quality of innovation circularity and its respective diffusion potential. To develop, test, and refine our framework—the “Circular Decision-Making Tree”—we synthesized insights from existing frameworks and conceptually integrated these with our understanding of transition theory and the circular economy. We then verified the internal logics and applicability of the framework in a series of usability workshops across four application contexts (Netherlands, Brazil, UK, and South Africa) with feedback from a total of *n* = 50 stakeholders from policy, practice, and academia. We critically discuss the application potential as well as the limitations and describe implications for future research to further validate the framework’s logics and operationalization.

## Introduction

### Circular Economy Context

In response to ever-increasing waste production and interrelated socio-environmental challenges, governments, businesses, and scholars have begun to embrace and support the concept of a circular economy (CE) [1]. The CE is a radically different economic paradigm that prioritizes the reduction of raw material extraction through value retention and regenerative design [2, 3]. The underlying purpose of adopting CE practices is to ultimately reduce virgin material consumption, eliminate waste, and decouple growth from material use [4–6]. Thus, not all movements within circularity contribute equally (or at all) to accelerating the desired transition to CE. Because a core value of CE is highest value preservation [7], it is possible that a circular innovation may contribute to the acceleration of CE—but at a low magnitude, relative to an alternative innovation that scores higher in a hierarchy of CE value retention options. Furthermore, the waste-resource paradox (WRP) occurring in the transition zone between a linear and a circular economy [8] elucidates that closing a loop through waste-based innovation [9] and turning a waste into a resource *may reinforce undesirable linear pathways* by incidentally creating a demand for said waste—thereby reinforcing its production, rather than deinstitutionalizing and breaking down its comfortable position in the supply chain [8].

Strategizing for and prioritization of higher-quality circularity proposals, solutions, and implementations may help to avoid low-value and low-impact CE solutions. Yet, even at the international EU level, “of the 36 CE Green Deals and 32 CE Best Practices, almost all aim at increasing recycling… Recycling, and low-grade recycling in particular, is still very much a linear solution. In addition to aiming for less resource consumption and waste generation, it is also important for a circular economy to focus on creating less environmental impact (including more value for ecology), and generating more added value for the economy” ([10], p. 39). This indicates that, despite existing efforts in science and practice, the vision of a circular economy is not yet translating into broader action to transition.

### Transition Theory

One central challenge in transitioning to CE is aligning short-term actions with long-term visions. In this case, the field of sustainability transitions may serve as appropriate research for guiding the convening of actions and visions. Specifically, transition management uses sustainable development as a guiding principle and relates to fundamentally new governance approaches [11]. While attempts at transitioning from a linear to a circular economy are being made, fundamental change requires a clear vision of both the future goals and the inherent tradeoffs. In transition research, we speak about a regime, or paradigm shift: a fundamental and structural change in the incumbent cultures, structures, and practices [12]. Transition literature describes regimes as path-dependent by nature, meaning that current decisions within an incumbent context are made based on previous decisions in that context and are not independent. The path dependencies are “inevitable, because of sunk investments, benefits of scale, and the co-evolutionary dynamic within a regime. But such path dependencies over time ultimately imply the inability to change beyond optimization, hence causing systemic tensions and problems” [13], p. 605). However, these incremental improvements often embed the assumption of continuing the current regime (and therefore, may lead to fostering existing aspects of the current regime). Dominant forms of policy and management are currently mostly prioritizing optimization—incremental improvement in the current regime—thereby adding to the lock-in of the current systems (ibid). To transition to a circular economy, we argue that transformative—i.e., fundamentally different—innovations and policies must be supported.

Alternatives to the regime are described as niches—in this case, circular innovations or policies. When these are scaled, replicated, or embedded into other contexts, this is known as diffusion [14]. Embedding refers to the adoption and integration of an innovation into existing institutions or regulations, translating addresses the process through which constitutive elements are replicated and reproduced elsewhere; scaling refers to the internal development and growth of niche innovations to a larger scale [15]. Based on the concept of the WRP—which describes the paradox in the “transition zone” between a linear and circular economy, wherein a material can be considered a waste and a resource at the same time, and cautions against related dilemmas that may ensue from using waste as materials for production [8]—we propose a new framework for decision-making in the context of transitioning to a CE. In this paper, we describe a new logic for operating decisions that may lead to more informed decisions towards circularity.

### Complexity of Circular Decision-making

Despite stated ambitions at various geographical scales to become more or fully circular, the outcomes fall short of the visions [10, 16–18]. We hypothesize that this is rooted in a fundamental problem observable in current practices of decision-making: often, decisions are made based on linear decision-making principles. Many forces and pressures influence decisions made on the CE—risk-aversion, stranded assets in linear business cases, path dependency of existing practices, locked-in institutions, and market fluctuations—which may prove difficult to penetrate or circumvent. However, another key challenge for amplifying the transition to CE is the still apparent lack of circular oriented governance and decision-making [19]. We posit that a particular obstacle that prevents sound circular decision-making (CDM) is that stakeholders dealing with CE may overlook the different quality levels of contributions to CE—or may lack decision-making support to navigate start-to-finish selecting the most impactful circular innovation to allocate resources to. Brown and colleagues support our argument, pointing to the current challenges of aligning circular innovation partners upon a shared circular purpose and the need for “developing a circular oriented value capture model focused on collective outcomes” ([19], p.13).

Funding and policy support for circular innovation is often disproportional to the potential contribution of the innovation towards the transition to a circular economy—meaning, the innovations with the highest potential for circular impact are not necessarily the ones that receive the most support. The current way of decision-making can be counter-productive to CE because of its support for incremental innovation [20]. For example, accelerating and scaling up an innovation that uses a waste as an input to the business model further ingrains the production of this waste in the economy. “Business-as-usual” linear pathways are created through “sustainable” innovations that fit within this scheme and thereby further reinforce these existing path dependencies—meaning, while incremental change may offer small gains in sustainable practices, its adoption reinforces the current way of operating and presents another barricade for transformative innovation to overcome. This indicates a need for a change towards a different type of decision-making logic.

The main objectives of our work are to formulate a decision-making logic that helps in taking decisions, considering the quality of innovation circularity and its respective diffusion potential. We also aim to operationalize and verify the framework with a case illustration and by testing the applicability with scientists and practitioners, considering political and cultural context variations, as well as to present this logic in a way that it supports learning-by-doing and reflexivity.

### Existing Tools and Current Gaps

Some related tools have been developed for predicting or informing decisions with an environmental impact, but none currently exist to assist practitioners in *navigating* their decisions operationally. These static models or schemes often capture only a moment in time and consider only a single factor of a decision as the basis for evaluation (e.g., the waste hierarchy); use inconsistent categorizations and terminologies, causing confusion among actors (e.g., the R-imperatives); or give a deceivingly precise quantitative result—when in fact many assumptions and estimations are put into the model—and do not allow the decision-maker autonomous operation (e.g., life cycle assessment (LCA)). In the field of decision-making, the bounded rationalities and other challenges around environmental policy and practice uncovered in multi-criteria decision analyses (MCDA) lead to uncertainties, indicating a need for an input-responsive, flexible framework for decision-makers to reference and help elicit improved questions to ask and steps in the decision [21, 22].

This gap between science and implementation in practice calls for a resource productivity-oriented framework [23]. Yet, the results of Zolfagharian et al.—an important assessment of 217 transition studies through systematic review—found that: “While current transition research is relatively strong in explaining past transitions and case studies, it seems less strong in designing (practical) interventions” ([38], p. 11). To address the dilemmas in the transition to a circular economy raised by the WRP, and in response to the gaps in literature and calls from previous researchers in this paper, we offer a new circular decision-making logic for actors and organizations. To operate this logic, we have created an operational framework called the Circular Decision-Making Tree (CDMT). The CDMT builds on some of the existing commonly referenced frameworks as introduced in Table 1:

**Table 1:** Existing tools related to a circular decision-making logic, added value, and limitations

Tool/approach	Primary added value	Limitations
Waste hierarchy	• Widely supported guide for waste management that [24] • Prioritizes waste treatment options to reduce environmental impacts in preferential order [25]	• Offers limited specification, implementation of prevention, and guidance for choosing among the levels of the hierarchy [23] • May result in stimulating optimization of the reigning linear economy (vs. fundamental change necessary for a new circular paradigm)
R-imperatives	• Illustrate hierarchies of CE value retention [4] • Frequently referenced as the “how-to” of CE (ibid) • Highlight the idea of value preservation or resource value retention options [26]	• Numbers, sequence, and terminology of these R-imperatives are inconsistent across frameworks, countries, and supranational organizations like the EU, the UN, and the OECD [4] • Contradictory syntheses of the R-imperatives built into complex political decision-making processes [25, 26]
Life cycle assessment (LCA)	• Analytical tool that captures the overall environmental impacts of all the life cycle stages associated [27] • Highlights potential environmental tradeoffs from one phase of the life cycle to another, from one region to another, or from one environmental problem to another [28]	• Compares “either-or” decisions; generally not designed to help select from a larger pool of innovation possibilities [29] • Does not give guidance through various steps of decisions [30] • User must already understand the environmental translation of the output value impacts, as well as when and why it would be appropriate to apply this tool [28]
Multi-criteria decision analysis (MCDA)	• Tool to discover and measure decision-maker considerations about various (mostly) non-monetary factors to compare alternative courses of action [31]	• Aims to model and predict the behavior of decision-makers, but lacks the capacity to help stakeholder navigate decision-making processes in real time [32, 33]

Still, a number of scholars have stressed the lack of appropriate CE tools and a shared language, such as in the context of CE-inspired business model innovation [34–36]. We recognize both the value and limitations of the tools and frameworks in Table 1, and we build on these in the circular decision-making logic embedded into the CDMT that we offer in this paper. In the existing approaches, we note a lack of guidance among circularity levels, a scheme that encourages transformative practices over optimization, a shortcoming in clarity and consistency across a sustainability rhetoric, an assessment tool that does not require an extensive scientific background to understand, and a heuristic to aid in predicting decision outcomes. Thus, while significant research has been conducted in the fields of waste management, environmental assessments, and decision-making, there remains an interdisciplinary gap between science and practice.

Since we are still in a linear economy (LE) regime (despite increasing circular efforts), we often see decision-makers in CE taking the existing economy as the starting point and trying to incrementally improve upon that. Yet, this may become counter-transformative by enhancing path dependencies and lock-in of the LE. To break free from the currently prevalent path-dependent logic of decision-making common in business and policy, we argue in this paper to rather take the perspective of a radically different future with radically different assumptions that minimize extraction and consumption striving respect planetary boundaries—beyond which human perturbations risk destabilizing the earth system at the planetary scale [37]. For these reasons, we argue that there is a need to take on a different decision-making logic: a circular decision-making logic. This distinct logic is embedded in our CDMT framework, which evolves from addressing the WRP dilemmas in CE (for example, the unintentional reinforcing of linear pathways through attempts at circular innovation) and based on the identified need for a distinct type of circular decision-making. In the transition to a circular economy, we hypothesize that a tool offering navigation through circular decisions—based on this logic—can provide useful orientation on hierarchically preferable contributions to a CE and diffusion potentials, through its intended operational and applicable format across sectors and societal domains. Accordingly, this paper is guided by the following research question:

- What kind of new decision-making logic is needed to address tensions and dilemmas that actors face in the transition to a circular economy?

The rest of the paper goes as follows: in the “Methodological Approach” section, we describe our methodological approach. In the “CDMT Construction and Design: Theoretical Basis and Validity” section, we discuss the theoretical basis for the CDMT and its design; in the “CDMT Steps and Flow” section, we explain the CDMT’s logics. In the “Verification of the CDMT Logics” section, we report the feedback from the focus group workshops. In the “Discussion” section, we discuss the results of our study, including insights gained through the study, limitations of the study, and future research recommendations stemming from our work. Finally, in the “Concluding Remarks” section, we end with concluding remarks summarizing our work and offering a short reflection on its contribution to science and practice.

## Methodological Approach

To develop the CDMT, we first drew upon existing literature and frameworks, and we formulated an initial draft version of a circular decision-making framework. We then tested and refined this framework in a series of workshops and interviews, exploring the practical uses and added value for decision-making (rather than to find out whether it serves as a comprehensive algorithm). As our aims were more linked to bringing theory into action, we have selected a pragmatic qualitative methodology for our research. Pragmatism in transition research allows for more innovative research designs and methodologies for what fits best and posits that true theories are those which can successfully enable and support action [38], which matches with our research aim. Qualitative research has been deemed to be more suitable to handle heterogeneous and multi-level nature of transitions [39, 40], so we have selected this methodology. In our qualitative methods, we took a four-prong approach:

First, we conducted a literature review [38, 41] to understand the current state of transition to a circular economy and what was lacking in science and practice. To form a solid theoretical and applicable foundation for the circular decision-making logic and corresponding framework, we reviewed literature around circular economy, decision-support tools, circular frameworks, environmental assessment methods and models, and national and international waste directives. This created our problematization and motivation for a framework to support in circular decision-making. The theory and frameworks analyzed were incorporated into the design of the CDMT to increase its internal validity [42]. We developed the framework’s logic building primarily on transition research and transition management theory (see e.g., [43, 44] and incorporating tools stemming primarily from the field of industrial ecology, as outlined earlier in Table 1 (see e.g., [45, 46]).

Second, we designed the CDMT. The construction and design of the CDMT involved the following: (1) synthesizing existing frameworks such as the waste hierarchy [47, 48] and R-imperatives [4, 26], (2) embedding the tool in existing theory, such as strategic niche management (SNM [49, 50], the multi-level perspective (MLP) [51, 52], transition management (TM) [11, 12], and technical innovation systems (TIS) [53, 54], and (3) selecting a decision tree format [55, 56] to design our framework. We will explain how these frameworks relate to the CDMT with further details in the following section.

Third, we conducted two successive focus group workshops in the Netherlands in order to verify the operational logics of the framework with scientists and practitioners, guided by well-known approaches for focus group methodologies [57, 58]. This focus group workshop method was selected because “focus groups, together with other qualitative methods, provide researchers with additional means of acquiring rich, experiential feedback from service users. Moreover, the supportive, congenial, non-judgmental setting offered by the focus group enhances the likelihood of collecting the diverse and spontaneous opinions that elude the in-depth interview and the nominal group technique” ([59], p. 504). Informed consent was obtained from all participants to be recorded and to publish results in a scientific journal. The recordings of the workshops were saved and stored on a secure computer.

The workshops were conducted online and video recorded. They took place in April and September of 2020, respectively, and had a scheduled duration of 2.5 hours each. In our research, we adopted principles of collaborative knowledge production in participatory processes, so the workshops were not a unilateral source of data collection; but rather, they had an element of co-design with participants—wherein they critiqued and informed on the framework constructs. Thus, after the workshops, we made some minor adjustments to the original CDMT in line with participant feedback and finally created the refined CDMT presented in this paper. For example, this included moving the economic evaluation earlier in the decision-making logic of the tree to indicate more immediate consideration. After the workshops, results were also shared back to participants.

In the first workshop, seventeen stakeholders from policy, research, practice, and government participated in the two workshops. Here we unpacked implicit risks, hampering factors, tradeoffs, and organizational dilemmas that factor into circular decision-making. In the first workshop, we presented to the groups the premise for the CDM and CDMT, based on our project context and the WRP conceptual framework. The floor was opened for question-and-answer sessions, and then two breakout groups were formed in order to discuss and explicate particular tradeoffs and inconsistencies encountered in their experience from practice and literature. In the second workshop with similar participants (in terms of numbers and fields of expertise), we investigated the CDMT’s usefulness to stakeholders and the soundness of its internal logics. We exemplified the pathways of the CDMT stepwise, illustrated with a case on plastics. After the initial introduction to the tool, participants joined working groups to explore and discuss the tool individually and collectively. Strengths, weaknesses, and limitations of the presented framework were identified as well as key aspects of usability for different stakeholder groups were identified. Notes were taken to create informal transcriptions in the breakout groups by an assigned note taker and by us during the plenary for cross-referencing verification.

Fourth and lastly, we replicated these workshops in other global contexts to test and strengthen the reliability of the tool, and to explore its potential for application in multiple contexts. The CDMT was originally developed in and reflected upon in the Dutch context, as a part of the Waste FEW ULL project funded by NWO (Dutch National Science Foundation), the European Union’s Horizon 2020 program, and the Joint Programming Initiative (JPI) Urban Europe. Within this project context, we worked with project partners across three continents and four countries with the aim to “reduce waste inefficiencies”. In a collaborative undertaking with our international project partners in Bristol, UK; São Paulo, Brazil; and Western Cape, South Africa; we tested the logics and tool in each distinct geopolitical context with a group of local stakeholders in each location, led by our project partners in that region. These contexts were selected for their involvement through the Waste FEW ULL project, but importantly—also for their differences in and diversity of circularity challenges, material stocks and flows, and governance contexts, in order to see the generic ability of the decision-making logics to be robust in these different contexts.

To approach the development of a multi-contextual framework, we reflected upon stakeholder feedback from both the Global North and Global South, synthesizing and integrating the feedback from participants to improve the logics, usability, and mapping of the tool in their respective contexts. We created a replicable reporting template for consistency of results reporting across country contexts. Each of the four investigative groups from Global South and Global North reported answers through the reporting template, the results of which were synthesized into a table of international reflections on the circular decision-making logics and feasibility of application in practical contexts. A total of *n* = 50 stakeholders reflected on the internal circular decision-making logics and the applicability of the CDMT. In all contexts, a combination of researchers and practitioners working on topics related to the circular economy gave feedback on the logics and design of the CDMT. To guide the discussion, the following guiding questions were used as prompts, replicated in each of the four contexts:

- Are there uncertainties, paradoxes, and dilemmas of decision-making that you consider barriers in the transition to a circular economy? What examples have you come across in your work or other area of activities?
- Do you agree with the internal logics of the CDMT? (What would you add or adjust for better usability?)
- (How) and for whom could the CDMT’s procedural logics support decision-making?
- What (if any) is the added value of the CDMT in helping distinguish innovations with higher-level contributions to a circular economy?

## CDMT Construction and Design: Theoretical Basis and Validity

The CDMT presents a framework that is organized in three sections (operational, strategic, and reflexive). Within each section, we identify concrete steps and, accordingly, decisions that may lead to the best possible steps to support a transition to circular economy. In defining the steps of the CDMT, our theoretical assumptions underlying the framework were informed by aspects from the following theories: SNM [49, 50], the MLP [51, 52], TM [11, 12], TIS [53, 54], and the WRP [8]. We critically reflected upon existing tools presented in the literature such as the waste hierarchy [47, 48], the R-imperatives [4, 26], and environmental impact assessment (EIA) [60, 61]. The steps in the CDMT are based on a CDM logic, which is based on a new economy paradigm: wherein transformational decisions are made for a radical new way of operating in society, rather than operationalization of (i.e., improvement of the existing) the linear economy and its inherent processes. In line with two fundamental principles of CE, the CDM logic prioritizes first the least virgin material extraction from the earth possible, followed by a cascading order of highest possible value retention of materials already in the system. The decision-making framework presented is built with this CDM logic as its backbone, and is created for practitioners to reference, in order to ideally make stronger and more transformative changes.

The development of the CDMT is framed at least in part by theory from transition scholars [53, 54], who identify in their TIS theoretical framework different types of systemic problems that can block the anchoring or widespread adoption of innovations. For example, actors’ problems may be capacity related: actors may lack competence or capacity to learn or utilize available resources, to identify and articulate their needs, and to develop visions and strategies. Institutional problems may also be capacity related, for example, when the institutions themselves are weak. Interaction problems may be presence related, if interactions are missing because of a cognitive distance between actors: differing objectives, assumptions, capacities, or lack of trust. Infrastructural problems—referring to physical, knowledge, and financial infrastructure—may be quality related: when an infrastructure is inadequate or malfunctioning (ibid, [62]). Through the framework of the CDMT, we aim to address these actor-related, institutional, and infrastructural problems identified by these authors.

Also central in the development of the CDMT is TM, a field of theory describing a governance mode based on complexity and with the goal of navigating the governance of long-term change processes towards more sustainable societies [63, 64]. Transition management deals with “key elements related to long-term governance of complex societal processes: multi-actor, long-term goal setting, innovation, evaluation and adaptation and knowledge transfer and learning” ([11], p. 79). These elements are also key pieces of the CDM logic that deals with innovation and adaptation. TM—like circular decision-making—is a highly uncertain and sometimes chaotic process. Based on this understanding and grounded in this theory, the CDMT is not designed to be prescriptive in nature to calculate an exact output, rather, like TM, one of the CDMT’s primarily functions is as “an attempt made to link different actors and organizations with different time horizons, ambitions, and values… [and] a way of indirectly influencing, adjusting, redirecting, and guiding actions” (ibid, p. 79). The CDMT was designed to support in directing decision choices and to help in guiding action during circular decisions.

The framework guides decision-makers to consider options in the sequence from most circular to least circular. In this way, the framework takes advantage of the common tendency for decision-makers to not consider all options but to satisfy, that is, choose the first option that reaches a satisfactory aspiration level. In contrast, sequencing in a framework can be defined as “planning a sequencing or deciding how to select the next task” [65], p. 2). Searching through options in sequence from the innovation with the highest-quality circularity and diffusion potential increases the likelihood that a more circular option is chosen (as compared with the opposite or a random sequence), so we have incorporated this heuristic principle into our framework.

To maintain the tool’s straightforwardness while accounting for factors outside of its immediate focus, the CDMT directs the user to a complementary tool at a decision point when the expertise becomes out of the scope of the heuristic. For example, when the decision-maker is unclear on what would be a more sustainable material to substitute for the current waste stream, the user is directed to an LCA. When considering uncertainty in cost-effectivity, the user is directed to a related tool, e.g., a cost–benefit analysis (CBA). It is our opinion that referring to other frameworks such as these can allow the tool to keep its visual simplicity while offering important information beyond the CDMT’s main focus of differentiation, potential impact on the transition to a circular economy. The CDMT and embedded decision-making logic was designed to be generic, independent of certain sectors or material types, and applicable across various geopolitical boundaries. It outlines a multitude of possible decision point uncertainties, answers, and resulting pathways that an actor may come across when operating on circular decision logics to help decision-makers navigate the process from start to finish.

The framework is broken down into three main columnar sections, grouped according to logical chronological order of steps to consider during a circular decision:

### “Operational” CDMT Column 1

The first column addresses the chronologically first and “operational” aspect, i.e., the initial innovation conceptualization, design, and production before potential scaling. The tool is designed so that the higher up on the tree the innovation ranks, the higher circularity potential it has. The framework bases its first column on other established circular economy tools, such as the waste hierarchy and R-imperatives discussed in the “Introduction” section. The CDMT matches these tools in indicating the same optimal result and similar preferences of cascading circularity quality. These other tools only miss indicating directionality in decision choices—here, the CDMT directs the user to incrementally consider the possibilities from the best option down, rather than simply “improving” environmental desirability from the bottom-up. We integrated this highly relevant aspect based on conceptual research that described the importance of preventing undesirable and unsustainable dilemmas that may result from turning a waste into a resource, described in the literature as the WRP [8]. Once a choice between circular innovations is made, the user advances to the second stage.

### “Strategic” CDMT Column 2

The second and “strategic” column prompts the decision-maker to examine the selected circular innovation’s diffusion potential, going beyond considerations of the innovation in isolation, and it focuses on the selected diffusibility and scalability potential. The CDMT encourages the decision-maker to consider deeper questions about the innovation’s scalability, replicability, and diffusibility: important factors into an innovation’s potential impact on circular economy beyond the local. This important component includes considerations of economies of scale, capital gains and financial feasibility, and the possibility of scaling in another cultural, political, social, or economic context. The CDMT’s second column’s theoretical underpinning stems from transition sciences: particularly, strategic niche management and the multi-level perspective, and with considerations from innovation diffusion theory as related to sustainability transitions [66–68]. The “strategic” column, as the name indicates, mirrors principles of strategic niche management [49, 50]. This is foundational literature describing the process for scaling up or diffusing a niche innovation to a regime level (the use of which concepts inherently embeds the MLP in our theoretical framework. In SNM, first, an innovation is chosen (akin to our column 1). Then, the environment for diffusion is examined and selected, similar to the column 2 constructs of the innovation scaling up in its current or another settings. Finally, the implementation is planned, examining resources in possession and needed, likening to the other constructs from column 2 that examine cost-effectivity and the ability to acquire additional resources. The steps of the strategic column of the CDMT are also echoed more recent literature, for example, von Wirth et al. [15]’s research on the types of diffusion, including scaling (vertical diffusion) by growing internally or translating (horizontal diffusion) in other contexts. In this way, validity is taken into account with the constructs of the strategic column by relating them to existing transition theory. In our research, we build on this and add to it by offering some potential answers or recommendations to alternative and complementary tools, to address some of the critiques and limitations of these theoretical frameworks.

### “Reflexive” CDMT Column 3

Because of the importance of reflexive learning in the continuous progression towards a circular economy, the third and final “reflexive” column prompts the user to evaluate and monitor the impact of the innovation selected. This includes identifying key factors and indicators to evaluate the impact of the circular innovation. After this assessment, the user may repeat the analysis for further material and energy flows. This evaluation and monitoring step brings the decision process full circle, concluding with a component of reflection for future learning. Having taken a transition lens in our paper and to construct the CDMT, we have assured for the validity of the final reflexive column by embedding these final constructs in foundational transition management literature. It is well-established in TM theory that continuous evaluation and monitoring is a vital part of the transition process [63, 69], and for this reason, it forms a necessary core part of the CDM logic as well.

How should we evaluate and monitor then? One prominent tool from industrial ecology is the environmental impact assessment, which has been shown to be helpful in decision-making and is defined as “the process of identifying, predicting, evaluating and mitigating the biophysical, social and other relevant effects of proposed development proposals prior to major decisions being taken and commitments made” [61]. Appropriately, we incorporated related elements of the EIA into the final constructs of the CDMT: “The identification of the main impacts brings together the previous steps with the aim of ensuring that all potentially significant environmental impacts (adverse and beneficial) are identified and taken into account in the process… The prediction of impacts aims to identify the magnitude and other dimensions of identified change in the environment with a project/action, by comparison with the situation without that project/action… Auditing follows from monitoring. It can involve comparing actual outcomes with predicted outcomes, and can be used to assess the quality of predictions and the effectiveness of mitigation. It provides a vital step in the EIA learning process” ([60], p. 5). Yet, an EIA is traditionally a quite formal, large-scale (e.g., bridge construction), and often rigid impact assessment. Thus, we draw from this analysis structure, integrating some key components of the EIA in the CDMT, while offering a less rigid evaluation and monitoring process for the likely smaller-scale innovations that could be considered when applying the CDMT.

## CDMT Steps and Flow

**Fig. 1:**
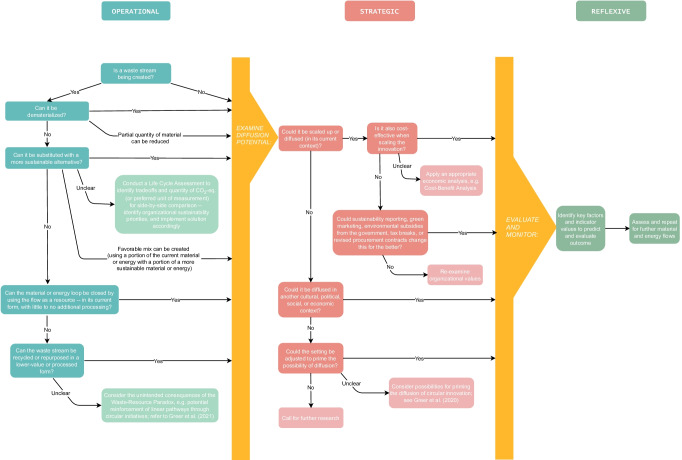
The Circular Decision-Making Tree (CDMT)

In this section, we explain the steps and flow of the CDMT, as well as how a decision-maker might walk through the framework to select a circular innovation to support with the highest potential for impact. See Fig. 1 below to follow the illustration.

### “Operational” Column: Innovation Prioritization

#### 1) Dematerialization

When comparing existing or potential initiatives to support within the circular economy, one would address the possibility of dematerialization, i.e., reduction of the amount of material required for a product or process [70]. Dematerialization is positioned first in the CDMT because reducing material use to preemptively prevent waste production is overwhelmingly the most efficient pathway to overall waste reduction from a product or process [71–73].

#### 2) Material Substitution

In the hypothetical scenario that no projects or innovations related to dematerialization are proposed, the user consider the possibility of material substitution: substituting in a suitable alternative material with a lower negative environmental impact intensity while fulfilling the same function [74]. If the potential for better material substitution is unclear, the CDMT directs decision-makers to the appropriate tool for the situation to inform the decision at the current stage: in this case, an LCA. If neither is possible, a more favorable material mix—i.e., a combination of a partial amount of the currently used material and an environmentally favorable material—is then suggested by the CDMT.

#### 3) Material Recovery and Reuse

If material substitution is not a viable route, the next alternative following the flow of the CDMT is to close loops through (waste) material reuse. Second-hand material cascading is a valuable initiative temporarily, but it is not transformative if the material production remains the same. Optimization can improve the current linear system, but it does not create a fundamental change towards an institutionalized alternative practice—which is why it is positioned as a lower alternative in the tree.

#### 4) Cascaded Recycling, Open-Loop Recycling, or Down-Cycling

If none of the aforementioned options is considered viable possibilities within their context, only then should the lower-value repurposing (e.g., recycling) be selected as the path forward. These innovations are tricky and placed last because of the potential to reinforce demand for a waste described by the WRP, furthering lock-in and optimization of the linear economy regime in place.

### “Strategic” Column: Diffusion and Acceleration Potential

Once a proposed circular innovation is selected from the first column, “strategic” considerations for the proposed innovation’s potential for diffusion are necessary to examine to understand its potential for more widespread impact.

#### 1) Scaling or Diffusing in Current Context.

Here, capacities for and limits to growth are analyzed. In principle, the most desired innovation is the one with highest-quality circularity, paired with highest diffusion potential. The user would first consider diffusion potential in the innovation’s current context. In the case that constraining factors are too great to scale up in its current context, one would move to the next consideration: the same innovation, scaled in another context.

#### 2) Scaling or Diffusing in a Different Context.

Here we also consider availability levels of non-financial resources of the selected circular innovation, but in another context. Might another cultural, political, social, or economic setting allow for room for diffusion that the current context does not? The answer to this determines the next step taken.

#### 3) Adjusting the Settings and Conditions Within the Context to Favor Diffusion Potential

If more favorable contexts for diffusion of the selected circular innovation do not currently exist, might they be created or facilitated? In that case, the CDMT refers the user to 15 practices and principles for connecting niche innovation with regime organizations by Greer et al. [75]. For example, “empowering actors to pursue alternative sustainable pathways” and “top-down influencing” may be applicable in this case as well.

#### 4) Cost-Effectivity of Scaling

This step does include economic considerations to add a piece to the picture of the scalability on a larger level. It is not a comprehensive check on economic viability directly within the tool. That level of economic detail is outside the scope of the tool, whose primary focus is on the material and energy aspects of decision-making outcomes.

### “Reflexive” Column: Monitoring and Evaluation of Implementation

The last phase of the CDM logic is incorporated for thoroughness and to set a baseline for evaluation and monitoring of the intended and actual impact of the innovation. In this step, key factors and indicator values to predict and evaluate the outcome should be developed. After a predetermined set of time, e.g., 1 year, of implementation of the initiative or innovation in practice, the innovation’s true impact over time should be assessed based on the designated criteria. Finally, after evaluation of the particular material at hand, the circular decision-making logic indicates to assess and repeat the exercise for further material and energy flows in this and other sectors of interest.

## Verification of the CDMT Logics

Now that we have illustrated the circular decision-making logics, we shortly describe the state of CE in the different verification contexts and report on the results of the applicability workshops.

### CE in International Contexts

As part of the research project Waste FEW ULL, we teamed up with international colleagues to conduct workshops and group interviews with stakeholders in their local contexts addressing waste inefficiencies and circular economy in the Netherlands, UK, South Africa, and Brazil. These countries are all on different pathways towards circularity, as summarized below:

#### Netherlands

Among diverse countries that have committed to CE targets, the Netherlands is a prominent pioneer in the pushing of circular economy (taking substantial steps towards their stated goal of full circularity by 2050) [76]. As supported by reports from the Netherlands Environmental Agency, a more ambitious CE transition towards substantially lower resource and material consumption and less generation of waste should be “based on high-circularity strategies, such as smarter manufacturing and use of products, and extending the lifetime of products and product components. Recycling alone, and low-grade recycling in particular, is still closely related to a linear economy” ([10], p. 7). Despite the clear vision of preferred pathways in the Netherlands, circular targets are still failing to be met [18]. The struggle of a frontrunning country in circularity, like the Netherlands, to meet targets indicates that our current strategies in decision-making around amplifying the circular economy are not effective in a meaningful enough way.

#### UK

For the past 3–4 decades, the UK has been progressing in its efforts towards the transition to a circular economy; however, the policy translation of these ambitions differs between its four countries. Some academic institutions, think tanks, and leading businesses have built on the foundations provided by European policy to raise awareness of the circular economy concept, bringing a more holistic approach to the various interpretations of the CE discourse. Whereas initiatives in waste management policy here were formerly concerned with end-of-pipe solutions, there is a recent collective change in understanding that keeping resources in productive use is the responsibility of both the producer and consumer, in a systems thinking approach to the problem [77].

#### South Africa

In practice, large volumes of valuable resources are transported to landfill. Sorting does occur at landfill, but the externality cost and access to these resources are lost to local inhabitants. There are opportunities to improve circularity, but conventional practices dominate, and there appears to be limited scope in policy and practice to intervene. Local authorities are largely overwhelmed by the scaled volume of waste, are under-resourced, and do not have the capacity to change the course of a conventional approach to a more circular practice. A hindrance to circularity in the South African context includes the inability to implement policies and interventions to divert food waste and organics from landfill. It is a combination of many factors that fail to transform the current trajectory: lack of leadership; establishing policy adds benefit to the waste and recovery value change; lack of constructive and cooperative partnership between local authority, private sector, and non-government organizations [78, 79].

#### Brazil

In Brazil, there are some widespread grassroots initiatives but lacking infrastructure for sustainability transitions. Currently, it is important to invest time in informing decision-makers about waste reduction and management practices. From a national perspective, there is still a lack of dissemination of sustainable innovation-based practices and technology adoption, as well as an absence of funding incentives for small producers. Current living lab projects are interesting and helpful but have a limited timeline and therefore a limited impact. Professionals involved often lose information and do not have access to possible technologies that could be implemented. The interaction between academia and public sectors is far from perfect due to this lack of information between institutions [80, 81].

### Verification Insights

The results of the workshops and group interviews in the Dutch, UK, Brazilian, and South African contexts, designed to test and validate the CDMT’s reliability in multiple distinct contexts, offered feedback on the usability and internal logics of the decision tree. It was discussed if and how the circular decision-making procedural logics could support decision-making and its application contexts as a guiding scheme. These full results are detailed below in Table 2 and are summarized thereafter:

**Table 2:** Verification of the circular decision-making tree in four global contexts

CDMT verification			
Context	Participants involved	Valued functions/perceived assets of the framework	Critical reflection/feasibility
Netherlands *n* = 17	• Researchers in academia • Think tank/researchers in practice • Consultancy • Policymaking	• Accurately reflects decision points and uncertainties in practice (consultancy and policy) • Useful when communicating/interacting with suppliers, as a way to evaluate circularity impact with a common frame of reference • Flexible in ability to apply when drawing up contracts (project leaders could raise these issues and target specific questions to relevant people)• Challenges incrementalism and encourages more transformative decisions	• Tradeoffs (e.g., with energy) and further system changes to be expected • Architectural or urban design context it might be hard to ever say yes to the questions • Best applied at a high/management level • Outcomes directly leading to circularity are not guaranteed
UK *n* = 11	• Researchers in academia • Policy • Activism/non-profit • Civic entrepreneurship • Community co-operatives	• Emphasizes the hierarchy in often-overlooked differences in contribution to CE • Creates a “CE architecture”: ability to articulate between systems, boundary object role • Added value may also be theoretical, demonstrating the need for more inter-sectoral or collaborative decision-making that goes beyond individual organizations	• Single waste stream focus • Entropy increases challenges in using the tool, because dispersed or mixed waste streams will be harder to address, e.g., smart phones with many components • Assumes upscaling is a goal, which it may not always be
South Africa *n* = 4	• Municipal waste management • Provincial government • Policymaking • Living lab management and academia	• Directs decision-makers to collect more data or to be prompted for existing evidence • Alerts decision-makers to be more critical in the evaluation of the flow and implications • Provides understanding and awareness about mechanisms for implementation and upscaling • Very useful if stakeholders are involved in the process and are able of participate freely in the development of decisions	• Might be missing crucial steps that give or improve the evidence that is necessary for decision-makers • Could propose mechanisms for implementation and upscaling • What tools could be put in place to enable the “Further Analysis” section that could support an enabling process to map the acceleration potential
Brazil *n* = 18	• Policymaking • Food technical production • Ministry for Agriculture and Food Supply • Non-profit • Researchers in academia	• Fills a gap for a management tool lacking in current practices, especially at the ministry level • Offers alternative to current majority bottom-up, chaotic planning by integrating increased rationality and structure in problem-solving • Locates interested parties and plans the strategy for good management of processes by mapping all working fronts from the beginning • Supports public policymaking and implementation and increase better time management performance by structuring next steps in user’s current work	• Could be built upon and adapted for other links in the value chain • Effects of waste treatment and processing not captured • Future developments of the tool could include a typology of characterization of waste • Many decisions depend on the technological trajectory • Socio-political definition of responsibilities for waste disposal is a factor
*Across all contexts* *n* = 50	• All of the above	• Convincing consensus on structural logics (of a new perspective on sustainable practices and strategic planning) • Helps broaden vision outside daily practices, encouraging systems thinking outside one’s immediate sphere • Internal logics helps problem framing around the quality of circular interventions. Helps identify dilemmas and paradoxes • Follows a logical path and draws attention to upscaling (to know what technology should be used and the viability of the innovation) • Catalyzes important reflection exercises and discussion on circularity processes and principles at multiple layers of companies, between and within public departments	• Should be paired with cost/benefit analyses within the status-quo of markets (account for capital, value distribution, return on investment) • An accompanying sectoral example makes logics clearer • Does not account for regulatory patterns on waste treatment • Power relations between actors, managers, companies, and waste management contractors are large and diverse

In summary, the Dutch workshops indicated an accurate reflection of decision points in the CDMT, its challenging of incrementalism, and its usability in communicating across supply chains or when drawing up contracts—the lattermost particularly supported by Dyer et al. [82], who underscores the importance of common interests, values, and priorities that in turn can contribute to a participatory design of a circular economy. The UK workshops observed the heuristic as a “CE architecture,” with potential to act as a boundary object for the user to articulate between systems. In South Africa, it was noted to offer autonomy and facilitate co-creative decision-making at micro-levels, meso-levels, and macro-levels. The Brazilian stakeholders indicated potential as a management tool and as a complementary alternative to more common bottom-up approaches.

An important critical reflection related to economic influences not being at the forefront of the tool’s guiding logic, since all decisions in reality are embedded in market and socio-economic contexts that deal with tradeoffs and challenges. The CDMT in its current state also does not include the influence of regulatory patterns on waste treatment or power dynamics. These remarks should be further addressed in later versions of the tool, which would be interesting additions for future versions of the tool, but out of the scope of this research. In its current state, the participants in all four contexts indicated that the circular decision logics may aid in procurement decisions, create awareness in suppliers of such circularity questions, catalyze reflexivity about the diversity of circular options, and identify dilemmas while drawing attention to the impact of upscaling. According to the participants, the CDMT may be useful at multiple layers of society—providing a common understanding of shared values, more clarity on the shared meaning of circularity, and the implementation practices needed to select, support, and carry out circular initiatives within their sphere of influence—for firms and companies, industrial symbioses, general policy, and sustainable entrepreneurship. From the results of the workshops and interviews, we see certain functions of the tool as being validated in a promising way. At the same time, we consider the critiques based upon which we offer recommendations for the future in the following subsection.

## Discussion

### Reflections on Results

Through the participatory feedback from workshops, we learned that different actors were prone to follow the steps of the CDMT with differing degrees of rigidity. While some participants indicated that the framework functions literally to identify a circular innovation or policy to select based on the quality of circularity and diffusion potential, it was suggested by others in the workshops that it would serve best in practice as a discussion tool or meta scheme: to catalyze circular thinking within and across departments or supply chains, to help strategize innovative priorities, and to co-create or build discussions around the steps involved in circular decision-making posed in the tool.

Strengths of the tree named in the focus groups include revealing the trap often surrounding the Waste-Resource Paradox (when a demand is created for a waste, thereby reinforcing the waste production, and sometimes monetizing it), helping to avoid or prevent this reinforcement of waste production. The decision tree discourages these linear economy optimizations that may also reinforce the path dependencies and inertia for the current linear economy regime. The CDMT presents a structuring and logic of circular decision-making, which re-orients a decision-maker who might otherwise make decisions that optimize and reinforce linear economy. In this way, it is representative of a new logic and improved steps in circular thinking. The analysis of 126 studies on thematic research areas of circular economy by Hina et al. indicated that “time, manager’s interest, information and employees’ awareness have been recognized as resources for Circular Economy Business Model implementation” ([83], p. 11), a gap in knowledge about which the authors recommended be addressed. Based on the findings from our study, we suggest that the application and utilization of the CDMT in practice might be one answer to address this gap by increasing information exchange, aligning values, and raising awareness about the highest-quality contributions to a circular economy. The results of our workshops indicated that CDMT could also act as a boundary object for different actors in decision-making collectives, which may help reduce fragmentation across disciplines and create a common framing for scientific dialogue.

This exploratory study on the CDMT showed us that the tool is also not without its contestations. It does not offer a comprehensive economic analysis, as some participants from the workshops suggested should be the most important consideration. Interestingly, the most commonly used existing circularity frameworks also miss these economic aspects, so we consider the CDMT as the next developed step by helping to operationalize preferred pathways and addressing acceleration potential. One of the assumptions of the application of the CDMT is that the user is looking for the highest-quality circularity innovation. So, while the CDMT might assist in business model creation, it is not a recipe for this. It is designed to help the user who prioritizes circularity—and also does incorporate economic considerations—but it is not designed to select the most profitable innovation that may still be considered circular. We recognize that this may make it less popular in practice, in the still highly linear profit-driven economy regime. Still, as resources decline and virgin material costs rise, it may become ever more important to consider circularity as a top priority, and it could be economically beneficial over the long term to be a circular frontrunner in this regard.

Similarly, discussions on the processing of waste affecting social implications for the positive or negative, for example, were not addressed in this first version of the tool. Holtz et al. state that “a single model therefore can hardly achieve the goals of completeness and detailedness” ([84], p. 50). This is due to the tradeoff between generality and context specificity of real-world transitions in models and frameworks, identified for example in theoretical literature on transitions by McDowall and Geels [40]. The CDMT was designed to cut through complexity to assist and guide its users through circular decisions. In simplifying some aspects of the decision, some factors have been condensed or left out. This was an intentional decision during the design of the tree, because incorporating too much complexity into the tool would eventually render it unusable. This condensing and simplifying of included considerations are always necessary for the first versions of tools and frameworks [40, 85], so further variables or social considerations should be taken up in a future research agenda. To offer increased transparency, aspects that have been excluded from this first version of the framework can be found in the Appendix.

For example, chemical waste is not directly considered in the CDMT. We found this to be less directly relevant for this first, more general CDMT design. However, this might be a critical factor for those working in the chemical field. Thus, we direct users to first examine the tool and its related assumptions to determine if it is appropriate in their sector before implementing it in their field. We also believe that future researchers can build on our 1.0 version of the CDMT. This might include experimenting with more depth in the economic consideration of the tree, or dissecting the reflexive column of the tool: questioning in what contexts can key factors be identified, and examining if and how scenarios be constructed. Relatedly, we recommend researchers to test the CDMT in various sectors and fields, who may build on our work by adopting this general CDMT and adapting it to include pertinent variables to their respective fields. Future researchers might develop industry-specific adaptations of the CDMT answering, for example, what secondary resource use would help their particular industry co-evolve towards circularity and sustainability.

### Limitations and Potential for Future Research

Our research was not without its limitations. For example, the workshops were replicated in four different contexts—but due to external factors, they were not exact copies of each other, e.g., in number of participants. These factors included the COVID-19 pandemic (no traveling was possible, so we could not do the empirical work and on-site observations as we had hoped), language barriers (the Brazilian focus group workshop was conducted by our project partners in Portuguese, who also translated the CDMT to Portuguese for this purpose—we authors could not personally present in this setting because we do not speak Portuguese), and what can be named issues of social justice (actors in the Brazilian and South African contexts had primarily poor access to high-speed internet, which limited the number of possible participants). In line with our pragmatic approach, we do not make conclusive statements about the generalizability or applicability of the CDMT in multiple socio-geopolitical contexts. Yet, the results do present some evidence that the CDMT might be useful in various distinct contexts. Even with less than perfect replication of settings, we were able to gain local insight in various distinct contexts, which we believe still serves to improve the reliability factor of the CDMT.

In our analysis, we employed the human-as-instrument method—which we believe was preferable to a coding software, for the ability to pick up on crucial non-verbal communication—but there is the possibility for human error or bias to enter here. While we tried to be as objective as possible, it is important for the reader of the study to keep in mind. This can also be said for the influence of some participants on others during the focus groups: “Doubts exist about the extent to which both the moderator and the ‘group effect’ influence individual participation in a focus group discussion. A comparative advantage of the focus group, however, is its ability to enable researchers to identify quickly the full range of perspectives held by the respondents. Moreover, the interactional, synergistic nature of the focus group allows participants to clarify or expand upon their contributions to the discussion in the light of points raised by other participants, thus expanding on contributions that might be left underdeveloped in an in-depth interview” ([59], p. 504). In choosing the methods for our study, we expected that the results gained from a fruitful group discussion would outweigh the risks of this workshop approach.

The CDMT tool itself may have practical limitations, since some decision-makers might still not know the answers to some of the questions posed in the framework. Even when all choices and information are clear, decision-makers may still be unable to implement its logics in practice—based on path dependencies, vested financial interests, power dynamics, or contractual obligations. These dilemmas are characteristic of most desired sustainability transitions, and they are difficult to overcome. That said, what we offer in this paper is one tool that may assist decision-makers in more carefully considering and navigating through tensions and dilemmas that decision-makers in a circular context may experience. In its first form presented here, the CDMT may offer more conceptual insight and awareness about quality of circularity and diffusion potential. Additional contexts for applying the tool named in the workshops include group management decisions, conversation-starting across and within departments, and for adding transparency and value alignment throughout value chains—which may potentially help address some of the related barriers of transitioning to a circular economy.

## Concluding Remarks

In our research, we combined theory from the fields of circular economy, transition management, industrial ecology, and decision-making to form a framework for decision-making towards a circular economy. By arguing for a new decision-making paradigm and offering an operational tool for navigating this logic in policy, practice, and society, we aimed to narrow the gap between scientific literature and practice through the creation and design of the CDMT framework. The guiding CDMT scheme built around the CDM logic was developed to improve upon existing circularity frameworks like the waste hierarchy and R-imperatives by directing decision-makers towards preferred circular initiatives, incorporating considerations such as diffusion and scaling potential, and emphasizing monitoring and evaluation of the impact of the selected initiative after a period of its implementation. The CDMT was intended to help actors select between circular innovations or proposals through a hierarchical process addressing highest-quality circularity and diffusion potential, while allowing for autonomy and flexibility in the decision-making. Through a series of focus group workshops, we enriched the first version of the tool and validated its internal logics and design esthetic by testing it with stakeholders in various geopolitical contexts.

From the results of our exploratory study, we see the CDMT as a conceptual and operational contribution to the current scientific debate in the governance of transitioning to circular economy, and it may add to practice by offering an improved mapping of decisions and collaborative orientation in decision-making involving multiple actors or organizations. It is clear that there is a diversity of pathways for transitioning from linear to circular economy, and there are internal and external barriers to the transition to an economy where circular systems are the norm. In its current form, the CDMT may already help lessen the gap between science and policy by providing orientation during decisions concerning circularity, communicating a hierarchy of preferable contributions in varying impact levels to CE, drawing attention to dilemmas and uncertainties in circular decision-making, incorporating considerations of diffusion or acceleration potential, and stimulating self-reflection and debate across sectors and societal domains. In conclusion, we believe this exploratory study has filled its purpose in developing and testing a new CDM logic and related framework that add to science and practice, and we recommend that future versions of the CDMT be developed to highlight other dimensions of sustainability and sector-specific constructs, building on our original work.

## Data Availability

Recordings of the workshops have been saved into a data repository according to the data management plan and will be stored for 10 years.

## Appendix

CDMT figure assumptions.

- By “scaling,” we refer to human, material, and financial resources/capacity to scale (economies of scales, limits to growth, etc.).
- Energy requirements are implicitly but not explicitly weighed.
- There is not a huge tradeoff with energy use, chemicals, or other environmentally harmful manifestations of burden shifting.
- Social dimension of sustainable development is not integrated (e.g., implications for labor force).
- All other components/factors remain reasonably constant (e.g., enormous transportation demands are not needed to receive the new material substituted).
- Recycling and material processing cause material and energy losses.
- Distinction of technical versus biological cycles is not central.
- Questions of material purity and absence of toxicity are not explicitly addressed.
- Industry-specific critical sustainability challenges or material resource shortages should be developed in future industry-specific research.

## Abbreviations

DRIFT: Dutch Research Institute for Transitions
CE: Circular economy
WRP: Waste-resource paradox
EU: European Union
CDM: Circular decision-making
LCA: Life cycle assessment
MCDA: Multi-criteria decision analyses
CDMT: Circular decision-making tree
UN: United Nations
LE: Linear economy
CBA: Cost-benefit analysis

## References

[1] Geissdoerfer M, Savaget P, Bocken NM, Hultink EJ (2017). The circular economy–a new sustainability paradigm?. J Clean Prod.

[2] Blomsma F, Brennan G (2017). The emergence of circular economy: a new framing around prolonging resource productivity. J Ind Ecol.

[3] Ellen MacArthur Foundation. (2021). Accessed on 23 April, 2021, from: https://www.ellenmacarthurfoundation.org/

[4] Campbell-Johnston K, Vermeulen WJ, Reike D, Bullot S (2020). The circular economy and cascading: towards a framework. Resour, Conserv Recycl.

[5] Ghisellini P, Cialani C, Ulgiati S (2016). A review on circular economy: the expected transition to a balanced interplay of environmental and economic systems. J Clean Prod.

[6] Murray A, Skene K, Haynes K (2017). The circular economy: an interdisciplinary exploration of the concept and application in a global context. J Bus Ethics.

[7] Zink T, Geyer R (2017). Circular economy rebound. J Ind Ecol.

[8] Greer R, von Wirth T, Loorbach D (2021) The waste-resource paradox: practical dilemmas and societal implications in the transition to a circular economy. Journal of Cleaner Production 303:126831. 10.1016/j.jclepro.2021.126831

[9] Henry M, Bauwens T, Hekkert M, Kirchherr J (2020). A typology of circular start-ups: an analysis of 128 circular business models. J Clean Prod.

[10] PBL - *Planbureau voor de Leefomgeving* (Netherlands Environmental Assessment Agency) (2017) Integrale Circulaire Economie Rapportage 2017. Available online (English Summary): www.pbl.nl/sites/default/files/downloads/2021-pbl-icer2021_english_summary-4228_0.pdf

[11] Loorbach D (2007). Transition management. *New mode of governance for sustainable development*.

[12] Rotmans J, Loorbach D (2009). Complexity and transition management. J Ind Ecol.

[13] Loorbach D, Frantzeskaki N, Avelino F (2017). Sustainability transitions research: transforming science and practice for societal change. Annu Rev Environ Resour.

[14] Smith A, Raven R (2012). What is protective space? Reconsidering niches in transitions to sustainability. Res Policy.

[15] von Wirth T, Fuenfschilling L, Frantzeskaki N, Coenen L (2019). Impacts of urban living labs on sustainability transitions: mechanisms and strategies for systemic change through experimentation. Eur Plan Stud.

[16] PACE - Platform for Accelerating the Circular Economy (2021). *The Circularity Gap Report*. Circle Economy. Available online: https://drive.google.com/file/d/1MP7EhRU-N8n1S3zpzqlshNWxqFR2hznd/edit

[17] Towa E, Zeller V, Achten WM (2021) Assessing the circularity of regions: stakes of trade of waste for treatment. J Ind Ecol. 10.1111/jiec.13106

[18] Hanemaaijer A et al (2021) Integrale Circulaire Economie Rapportage 2021. Den Haag: PBL. PBLpublicatienummer:4124. Available online: www.pbl.nl/sites/default/files/downloads/2021-pbl-icer2021_english_summary-4228_0.pdf

[19] Brown P, Von Daniels C, Bocken NMP, Balkenende AR (2021). A process model for collaboration in circular oriented innovation. J Clean Prod.

[20] Ritzén S, Sandström GÖ (2017). Barriers to the circular economy–integration of perspectives and domains. Procedia Cirp.

[21] Kalbar PP, Karmakar S, Asolekar SR (2012). Selection of an appropriate wastewater treatment technology: a scenario-based multiple-attribute decision-making approach. J Environ Manage.

[22] Kalbar PP, Karmakar S, Asolekar SR (2016). Life cycle-based decision support tool for selection of wastewater treatment alternatives. J Clean Prod.

[23] van Ewijk S, Stegemann JA (2016). Limitations of the waste hierarchy for achieving absolute reductions in material throughput. J Clean Prod.

[24] Dijkgraaf E, Vollebergh HR (2004). Burn or bury? A social cost comparison of final waste disposal methods. Ecol Econ.

[25] Hultman J, Corvellec H (2012). The European waste hierarchy: from the sociomateriality of waste to a politics of consumption. Environ Plan A.

[26] Reike D, Vermeulen WJ, Witjes S (2018). The circular economy: new or refurbished as CE 3.0?—exploring controversies in the conceptualization of the circular economy through a focus on history and resource value retention options. Resour Conserv Recycl.

[27] Finnveden G, Hauschild MZ, Ekvall T, Guinée J, Heijungs R, Hellweg S, ... Suh S (2009). Recent developments in life cycle assessment. J Environ Manag, 91(1), 1-21 10.1016/j.jenvman.2009.06.018 19716647

[28] Curran MA (2014) Strengths and limitations of life cycle assessment. In *Background and future prospects in life cycle assessment* (pp. 189–206). Springer, Dordrecht

[29] de Haes HAU, Heijungs R, Suh S, Huppes G (2004). Three strategies to overcome the limitations of life-cycle assessment. J Ind Ecol.

[30] Finnveden G (2000). On the limitations of life cycle assessment and environmental systems analysis tools in general. The International Journal of Life Cycle Assessment.

[31] Huang IB, Keisler J, Linkov I (2011). Multi-criteria decision analysis in environmental sciences: ten years of applications and trends. Sci Total Environ.

[32] Groeneveld J, Müller B, Buchmann CM, Dressler G, Guo C, Hase N, ... Schwarz N (2017). Theoretical foundations of human decision-making in agent-based land use models–a review. Environ model softw, 87, 39-48

[33] Smith HR (1979) A simulator study of the interaction of pilot workload with errors, vigilance, and decisions. Document ID19790006598. Available online: https://ntrs.nasa.gov/api/citations/19790006598/downloads/19790006598.pdf

[34] Antikainen M, Valkokari K (2016) A framework for sustainable circular business model innovation. Technol Innov Manag Rev 6(7). 10.22215/timreview/1000

[35] Bocken NM, Bom CA, Lemstra H (2017, October) Business-led sustainable consumption strategies: the case of HOMIE. In 18th ERSCP Conference. Available online: www.researchgate.net/profile/Nancy-Bocken/publication/320443318_Businessled_sustainable_consumption_strategies_the_case_of_HOMIE/links/59e5a19e0f7e9b0e1ab22665/Business-ledsustainable-consumption-strategies-the-case-of-HOMIE.pdf

[36] Lewandowski M (2016). Designing the business models for circular economy—towards the conceptual framework. Sustainability.

[37] Steffen W, Richardson K, Rockström J, Cornell SE, Fetzer I, Bennett EM, ... Sörlin S (2015) Planetary boundaries: guiding human development on a changing planet. Science 347(6223). 10.1126/science.1259855 10.1126/science.1259855 25592418

[38] Zolfagharian M, Walrave B, Raven R, Romme AGL (2019). Studying transitions: past, present, and future. Res Policy.

[39] Andersson C, Törnberg A, Törnberg P (2014). Societal systems–complex or worse?. Futures.

[40] McDowall W, Geels FW (2017). Ten challenges for computer models in transitions research: commentary on Holtz et al. Environ Innov Soc Transit.

[41] Snyder H (2019). Literature review as a research methodology: an overview and guidelines. J Bus Res.

[42] Drost EA (2011). Validity and reliability in social science research. Education Research and perspectives.

[43] Elzen B, Geels FW, Green K (eds) (2004) System innovation and the transition to sustainability: theory, evidence and policy. Edward Elgar Publishing. 19–29

[44] Geels FW (2010). Ontologies, socio-technical transitions (to sustainability), and the multi-level perspective. Res Policy.

[45] Ayres RU, Ayres L (eds) (2002) A handbook of industrial ecology. Edward Elgar Publishing. 10.4337/9781843765479

[46] Deutz P, Ioppolo G (2015). From theory to practice: enhancing the potential policy impact of industrial ecology. Sustainability.

[47] Gharfalkar M, Court R, Campbell C, Ali Z, Hillier G (2015). Analysis of waste hierarchy in the European waste directive 2008/98/EC. Waste Manage.

[48] Pires A, Martinho G (2019). Waste hierarchy index for circular economy in waste management. Waste Manage.

[49] Kemp R, Schot J, Hoogma R (1998). Regime shifts to sustainability through processes of niche formation: the approach of strategic niche management. Technology analysis & strategic management.

[50] Schot J, Geels FW (2008). Strategic niche management and sustainable innovation journeys: theory, findings, research agenda, and policy. Technology analysis & strategic management.

[51] Geels FW (2002). Technological transitions as evolutionary reconfiguration processes: a multi-level perspective and a case-study. Res Policy.

[52] Smith A, Voß JP, Grin J (2010). Innovation studies and sustainability transitions: the allure of the multi-level perspective and its challenges. Res Policy.

[53] Bergek A, Hekkert M, Jacobsson S, Markard J, Sandén B, Truffer B (2015). Technological innovation systems in contexts: conceptualizing contextual structures and interaction dynamics. Environ Innov Soc Trans.

[54] Bergek A, Jacobsson S, Carlsson B, Lindmark S, Rickne A (2008). Analyzing the functional dynamics of technological innovation systems: a scheme of analysis. Res Policy.

[55] Priyam A, Abhijeeta GR, Rathee A, Srivastava S (2013). Comparative analysis of decision tree classification algorithms. International Journal of current engineering and technology.

[56] Quinlan JR (1996). Learning decision tree classifiers. ACM Computing Surveys (CSUR).

[57] Krueger RA (2014) Focus groups: a practical guide for applied research. Sage publications

[58] Parker A, Tritter J (2006). Focus group method and methodology: current practice and recent debate. International Journal of Research & Method in Education.

[59] Powell RA, Single HM (1996). Focus groups. Int J Qual Health Care.

[60] Glasson J, Therivel R (2013). Introduction to environmental impact assessment.

[61] IAIA (2009) What is impact assessment? Fargo, ND: IAIA. Available online: https://www.iaia.org/uploads/pdf/What_is_IA_web.pdf

[62] Wieczorek AJ, Hekkert MP (2012). Systemic instruments for systemic innovation problems: a framework for policy makers and innovation scholars. Science and public policy.

[63] Loorbach D (2010). Transition management for sustainable development: a prescriptive, complexity-based governance framework. Governance.

[64] Rotmans J, Kemp R, Van Asselt M (2001) More evolution than revolution: transition management in public policy. Foresight 3(1):15–31. 10.1108/14636680110803003

[65] Baker KR, Trietsch D (2013). Principles of sequencing and scheduling.

[66] Boons F, Montalvo C, Quist J, Wagner M (2013). Sustainable innovation, business models and economic performance: an overview. J Clean Prod.

[67] Loorbach D, Wittmayer J, Avelino F, von Wirth T, Frantzeskaki N (2020). Transformative innovation and translocal diffusion. Environ Innov Soc Trans.

[68] Rogers EM (2010). Diffusion of innovations.

[69] Kemp R, Loorbach D (2006). Transition management: a reflexive governance approach.

[70] Thomas VM (2003). Demand and dematerialization impacts of second-hand markets: reuse or more use?. J Ind Ecol.

[71] Bizcocho N, Llatas C (2019). Inclusion of prevention scenarios in LCA of construction waste management. The International Journal of Life Cycle Assessment.

[72] Cleary J (2014). A life cycle assessment of residential waste management and prevention. Int J Life Cycle Assess.

[73] Gentil EC, Gallo D, Christensen TH (2011). Environmental evaluation of municipal waste prevention. Waste Manage.

[74] D’Amico B, Pomponi F, Hart J (2021). Global potential for material substitution in building construction: the case of cross laminated timber. J Clean Prod.

[75] Greer R, von Wirth T, Loorbach D (2020). The diffusion of circular services: transforming the Dutch catering sector. J Clean Prod.

[76] Rijksoverheid (2021). *Nederland Circulair in 2050.* Accessed on 25 March, 2021, from: https://www.rijksoverheid.nl/onderwerpen/circulaire-economie/nederland-circulair-in-2050

[77] Hill J (2016). Circular economy and the policy landscape in the UK. In *Taking stock of industrial ecology* (pp. 265–274). Springer, Cham

[78] Mativenga PT, Agwa-Ejon J, Mbohwa C, Shuaib NA (2017). Circular economy ownership models: a view from South Africa industry. Procedia Manufacturing.

[79] Rodseth C, Notten P, Von Blottnitz H (2020). A revised approach for estimating informally disposed domestic waste in rural versus urban South Africa and implications for waste management. S Afr J Sci.

[80] Guarnieri P, Cerqueira-Streit JA, Batista LC (2020) Reverse logistics and the sectoral agreement of packaging industry in Brazil towards a transition to circular economy. Resour Conserv Recycl 153:104541

[81] Paes MX, de Medeiros GA, Mancini SD, de Miranda Ribeiro F, de Oliveira JAP (2019) Transition to circular economy in Brazil: A look at the municipal solid waste management in the state of São Paulo. Manag Decis 59(8):1827–1840

[82] Dyer M, Wu S, Weng MH (2021) Convergence of public participation, participatory design and NLP to co-develop circular economy. Circular Economy and Sustainability 1–18. 10.1007/s43615-021-00079-0

[83] Hina M, Chauhan C, Kaur P, Kraus S, Dhir A (2022). Drivers and barriers of circular economy business models: where we are now, and where we are heading. J Clean Prod.

[84] Holtz G, Alkemade F, Haan De F, Köhler J, Trutnevyte E, Luthe T … Ruutu S 2015 Prospects of modelling societal transitions: position paper of an emerging community, Environ Innov Soc Trans, 17; 41-58

[85] Scott JC (2008) Seeing like a state. In Seeing Like a State. Yale University Press. 80. 10.12987/9780300128789

